# Phospho-Form Specific Substrates of Protein Kinase B (AKT1)

**DOI:** 10.3389/fbioe.2020.619252

**Published:** 2021-02-03

**Authors:** McShane McKenna, Nileeka Balasuriya, Shanshan Zhong, Shawn Shun-Cheng Li, Patrick O'Donoghue

**Affiliations:** ^1^Department of Biochemistry, The University of Western Ontario, London, ON, Canada; ^2^Department of Chemistry, The University of Western Ontario, London, ON, Canada

**Keywords:** genetic code expansion, AKT1, kinase, phosphoserine, tRNASep, PDK1

## Abstract

Protein kinase B (AKT1) is hyper-activated in diverse human tumors. AKT1 is activated by phosphorylation at two key regulatory sites, Thr308 and Ser473. Active AKT1 phosphorylates many, perhaps hundreds, of downstream cellular targets in the cytosol and nucleus. AKT1 is well-known for phosphorylating proteins that regulate cell survival and apoptosis, however, the full catalog of AKT1 substrates remains unknown. Using peptide arrays, we recently discovered that each phosphorylated form of AKT1 (pAKT1^S473^, pAKT1^T308^, and ppAKT1^S473,T308^) has a distinct substrate specificity, and these data were used to predict potential new AKT1 substrates. To test the high-confidence predictions, we synthesized target peptides representing putative AKT1 substrates. Peptides substrates were synthesized by solid phase synthesis and their purity was confirmed by mass spectrometry. Most of the predicted peptides showed phosphate accepting activity similar to or greater than that observed with a peptide derived from a well-established AKT1 substrate, glycogen synthase kinase 3β (GSK-3β). Among the novel substrates, AKT1 was most active with peptides representing PIP3-binding protein Rab11 family-interacting protein 2 and cysteinyl leukotriene receptor 1, indicating their potential role in AKT1-dependent cellular signaling. The ppAKT1^S473,T308^ enzyme was highly selective for peptides containing a patch of basic residues at −5, −4, −3 and aromatic residues (Phe/Tyr) at +1 positions from the phosphorylation site. The pAKT1^S473^ variant preferred more acidic peptides, Ser or Pro at +4, and was agnostic to the residue at −5. The data further support our hypothesis that Ser473 phosphorylation plays a key role in modulating AKT1 substrate selectivity.

## Introduction

Protein kinase B (AKT) belongs to the AGC family of serine-threonine kinases (Manning and Toker, 2017). In mammals, three *Akt* genes encode the AKT isozymes AKT1, AKT2, and AKT3. The three isozymes display high sequence identity and similar substrate specificity (Manning and Cantley, 2007; Manning and Toker, 2017). The AKT isozymes share the same fundamental structure consisting of four distinct domains: an N-terminal regulatory pleckstin homology (PH) domain, an unstructured linker region connecting the PH domain to the serine/threonine-specific kinase domain; and a final C-terminal domain often referred to as the hydrophobic motif, which is responsible for modulating the activity of AKT1 (Franke, 2008; Balasuriya et al., 2020). AKT1 is a key regulatory kinase that transduces signals through the phosphoinositide 3-kinase (PI3K)/AKT cell-signaling cascade that controls cell growth and survival (Burgering and Coffer, 1995). The PI3K/AKT1 pathway is one of the most commonly deregulated pathways in human cancer (Altomare and Testa, 2005; Dai et al., 2005). In fact, AKT1 is hyper-phosphorylated and overactive in >50% of human tumors (Agarwal et al., 2013; Spencer et al., 2014; Manning and Toker, 2017). Elevated AKT1 phosphorylation status is linked to poor clinical prognosis (Dai et al., 2005; Suzuki et al., 2010; Antonelli et al., 2012).

### AKT1 Activation

Activation of AKT1 is a multi-step process that involves a carefully orchestrated series of cellular translocation and post translational modification events (Manning and Toker, 2017). Indeed, serine/threonine phosphorylation (Alessi et al., 1996; Balasuriya et al., 2018a,b, 2020), methylation (Guo et al., 2019; Wang et al., 2019), ubiquitination (Yang et al., 2009; Cederquist et al., 2017; Wang et al., 2018), and proline hydroxylation (Guo et al., 2016) have been shown to regulate the activation or to modify the enzymatic activity of AKT1. Among these post translational modifications, serine/threonine phosphorylation has been studied most extensively (Alessi et al., 1996; Kunkel et al., 2005; Manning and Cantley, 2007; Manning and Toker, 2017; Balasuriya et al., 2018a, 2020). Phosphorylation of the threonine residue at position 308 (T308) in the activation loop of AKT1 is both necessary and sufficient to achieve maximal AKT1 signaling in cells (Balasuriya et al., 2018a) and oncogenic transformation (Hart and Vogt, 2011), however, a second phosphorylation of the serine residue at position 473 (S473) in the C-terminal hydrophobic motif of AKT1 leads to increased AKT1 catalytic activity (Alessi et al., 1996) with certain substrates (Balasuriya et al., 2020).

**Figure 1:**
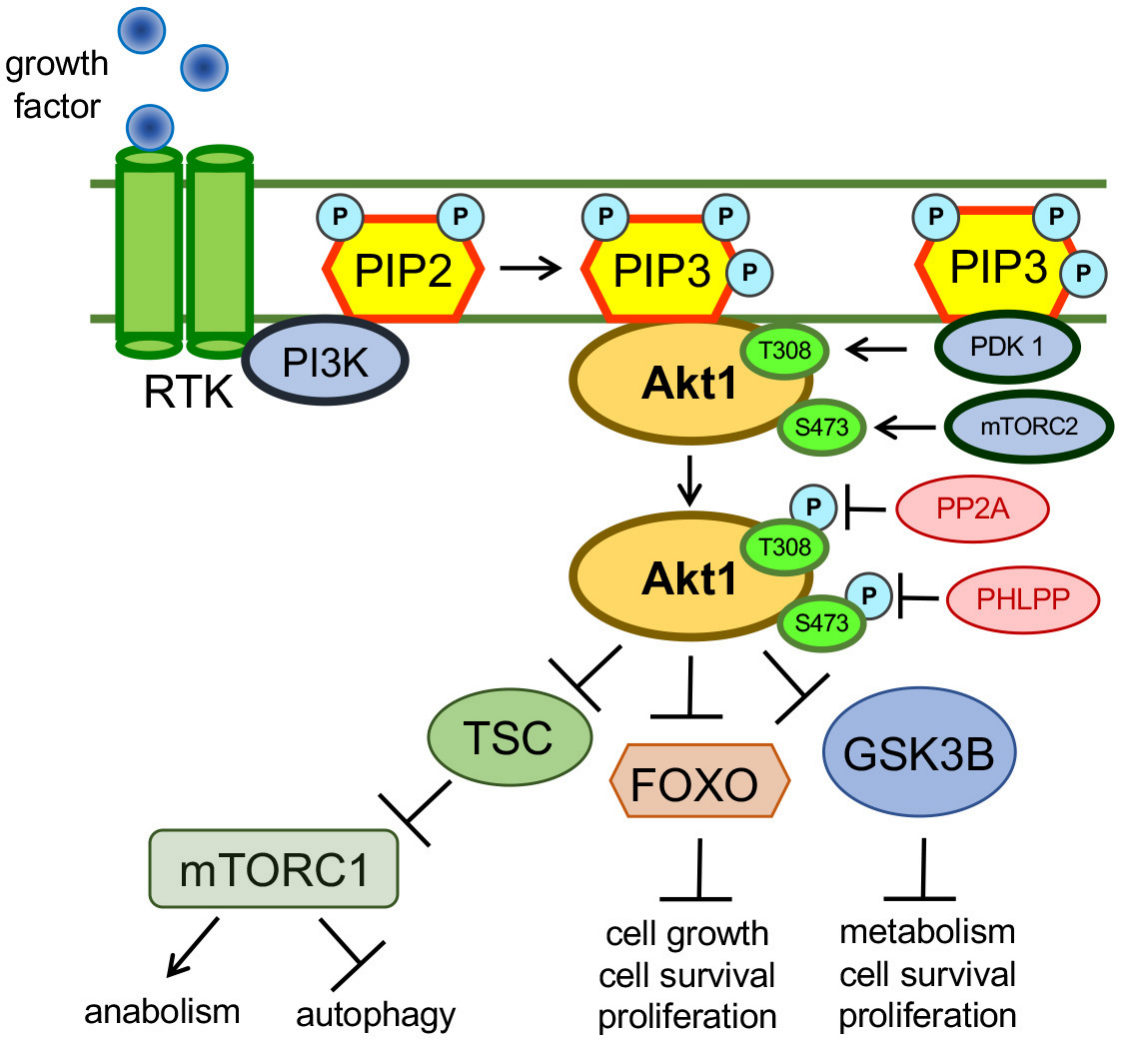
Growth factor mediated activation of AKT1. AKT1 is activated in response to growth factor mediated stimulation of the PI3K pathway. Activated AKT1 promotes an oncogenic cellular phenotype that exhibits inhibited autophagy and apoptosis pathways as well as activated cellular growth, proliferation and anabolic pathways. AKT1 targets many cellular proteins and the complete catalog of AKT1 substrates is unknown. The above examples show how AKT1 inhibits autophagy *via* the phosphorylation of the tuberous sclerosis complex (TSC). Through intermediates not shown, phosphorylated TSC can no longer maintain inhibition of mTORC1, so mTORC1 activity is simulated downstream of active AKT1 (Saxton and Sabatini, 2017). AKT1 also inhibits apoptosis and promotes cellular growth through phosphorylation and subsequent inhibition of the tumor suppressors FOXO and GSK-3β (van der Vos and Coffer, 2010; Kaidanovich-Beilin and Woodgett, 2011).

**Figure 2:**
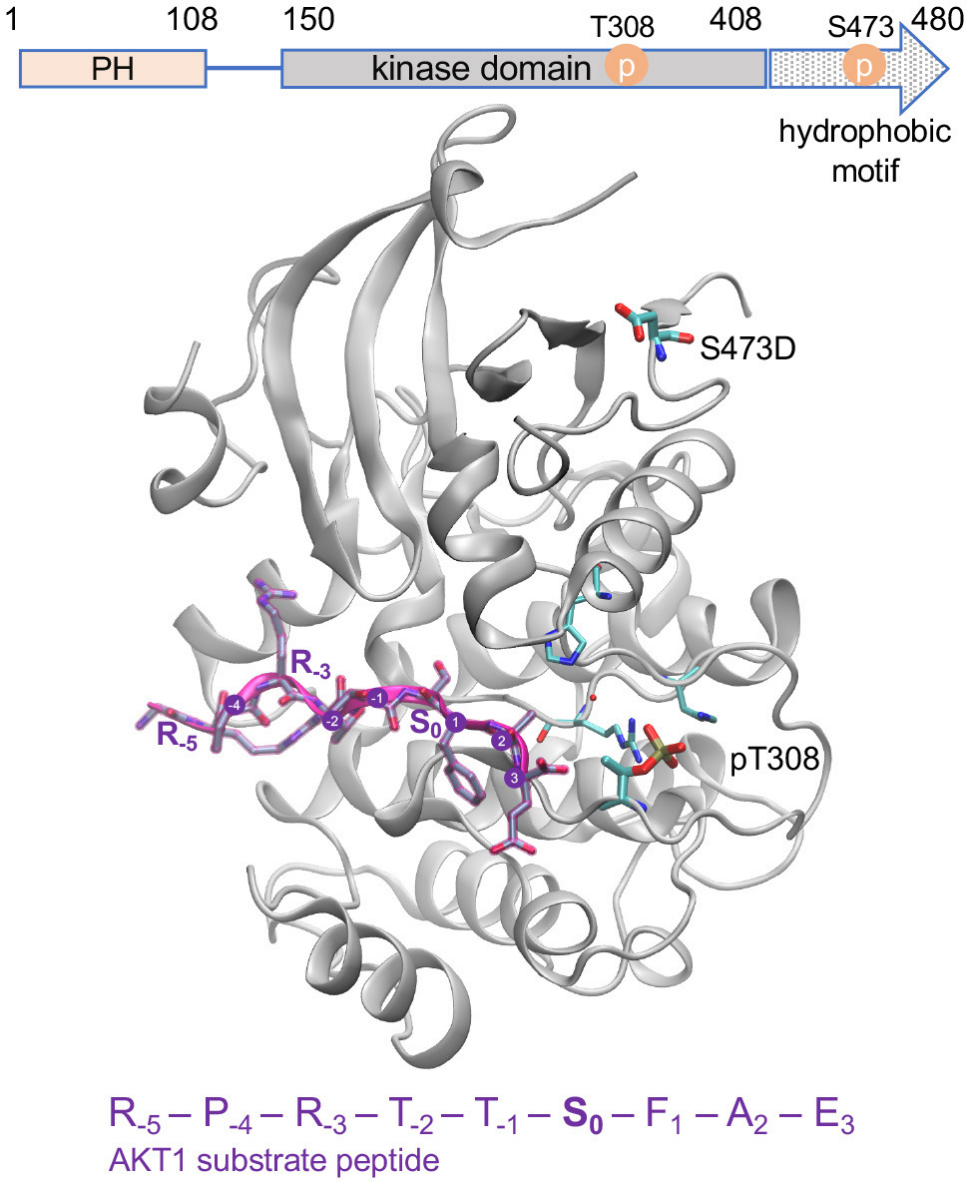
Active AKT1 bound to substrate peptide. The AKT1 structure [PDB code 3CQU (Lippa et al., 2008)] shows the kinase domain and hydrophobic motif of AKT1 (gray cartoon, residues 144–480) bound to a substrate peptide (purple). The domain organization of AKT1 is shown above. The GSK-3β derived peptide sequence is indicated, and positions surround the phosphorylation site (S_0_) are annotated. The phospho-accepting site (S_0_) is oriented in toward the active site. The structure contains pT308 and a mutation at the S473 site to aspartic acid (D). The key activating phosphorylation site, T308, is intricately connected to the active site and peptide binding site of the enzyme while the S473 site, located in the C-terminal hydrophobic motif, is distant from the active site.

In cells, the phosphorylation of AKT1 at positions T308 and S473 results from insulin or growth factor stimulation of receptor tyrosine kinases and G-protein coupled receptors on the cell surface (Alessi et al., 1996; Vanhaesebroeck et al., 2010). Growth factor stimulation promotes plasma membrane recruitment and subsequent activation of members of the class I PI3K family (Figure 1). Following activation by receptor kinases, PI3K phosphorylates phosphatidylinositol-4,5-bisphosphate (PIP2), converting PIP2 into phosphatidylinositol-3,4,5-triphosphate (PIP3) (Whitman et al., 1988). PIP3 acts a scaffold and forms an interaction with the PH domains of various proteins, including AKT1 and phosphoinositide dependent kinase 1 (PDK1), an AGC family kinase that phosphorylates AKT1 at T308. The interaction between PIP3 and the AKT1 PH domain causes AKT1 to adopt a “PH-out” conformation, relieving AKT1 of the auto-inhibitory effect of its own PH domain (Parikh et al., 2012). The “PH-out” conformation of AKT1, along with its proximity to PDK1, mediated by their interactions with PIP3, allows PDK1 to activate AKT1 by phosphorylation at T308 (Alessi et al., 1996; Stokoe et al., 1997). AKT1 is phosphorylated at S473 by the mechanistic target of rapamycin complex 2 (mTORC2) (Sarbassov et al., 2005; Figure 2). mTORC2-mediated phosphorylation of AKT1 at S473 is itself dependant on the contribution and activity of many protein factors, including Protor (Pearce et al., 2007), Sin1 (Yang et al., 2006), and TSC2 (Huang and Manning, 2009; Liao and Hung, 2010) that function to stabilize mTORC2. AKT1 activity is down-regulated (Kunkel et al., 2005) *via* de-phosphorylation at T308 by protein phosphatase 2A (PP2A) (Kuo et al., 2008) and at S473 by PH domain leucine-rich repeat protein phosphatases (PHLPP) (Brognard et al., 2007).

### Therapeutic Relevance of AKT1

The AKT1 signaling pathway is a useful prognostic marker of many cancers as well as a promising target for therapies (Suzuki et al., 2010; Antonelli et al., 2012; Agarwal et al., 2013; Spencer et al., 2014). Targeting the AKT1 pathway has been the subject of over 300 clinical trials (Testas et al., 1989; Nitulescu et al., 2016). The majority of these clinical trials investigate small molecule inhibitors (Mattmann et al., 2011) as potential tools which can be used to inhibit or reduce AKT1 hyper-activity. Unfortunately, the structural and functional similarity of AKT1 to its isozymes and other members of the AGC-kinase family has made it difficult to solely target AKT1 without also inhibiting additional kinases and causing unwanted side effects in clinical trials (Cheng et al., 2005).

### AKT1 Signaling Network

Ultimately, the cellular consequences of AKT1 activity are determined by the profile of substrates that it either activates or deactivates *via* phosphorylation in any given cell (Manning and Toker, 2017). In order to identify new therapeutic targets for AKT1-dependent cancers, it is important to identify the complete catalog of AKT1-dependent substrates. By expanding the scope of pathways regulated by AKT1, new routes will emerge to inhibit specific interactions between AKT1 and downstream substrates. Some AKT1 substrates are causative agents in pathologies such as cancers (Gonzalez and McGraw, 2009) or diabetes (Hers et al., 2011). For example, in a study of a rat model of the proapoptotic protein Par-4, it was found that AKT1 was directly responsible for the inactivating phosphorylation of Par-4, which inhibited apoptosis and promoted oncogenesis (Goswami et al., 2005).

AKT1 has >150 reported substrates (Manning and Toker, 2017) involved in a wide variety of cellular events, including signal transduction, metabolism, cell-cycle regulation, transcription regulation, proliferation, and angiogenesis. The level of certainty that all of these substrates are *bona fide* AKT1 targets is substantiated by differing levels of evidence. All substrates adhere to some degree to the AKT1 phosphorylation motif. Pioneering efforts with peptide arrays defined the consensus AKT1 target motif as R_−5_X_−4_R_−3_X_−2_X_−1_S/T_0_φ_1_, where X represents any amino acid, φ represents any bulky aromatic residue and subscript numbers refer to the residue's position relative to the phosphorylated residue S_0_ or T_0_ (Obata et al., 2000). Many of the now known AKT1 substrates were originally validated by *in vitro* kinases assays, including well-established AKT1 targets FOXO1A (Rena et al., 1999), GSK-3β (Hornbeck et al., 2004), and WNK1 (Vitari et al., 2004). Traditionally, *in vivo* studies in cells or mouse models are used to further validate reported AKT1 substrates. Homozygous knock-in mouse strains that either lack PDK1 expression, PDK1(-/-) (Williams et al., 2000), or that express a PDK1 variant that is unable to activate AKT1 (McManus et al., 2004), have been used in combination to demonstrate the ability of AKT1 to phosphorylate PRAS40, FOXO1, FOXO3, GSK-3β, TSC2, and WNK1 (McManus et al., 2004).

### Investigating AKT1 Substrate-Specificity Using Genetic Code Expansion

**Figure 3:**
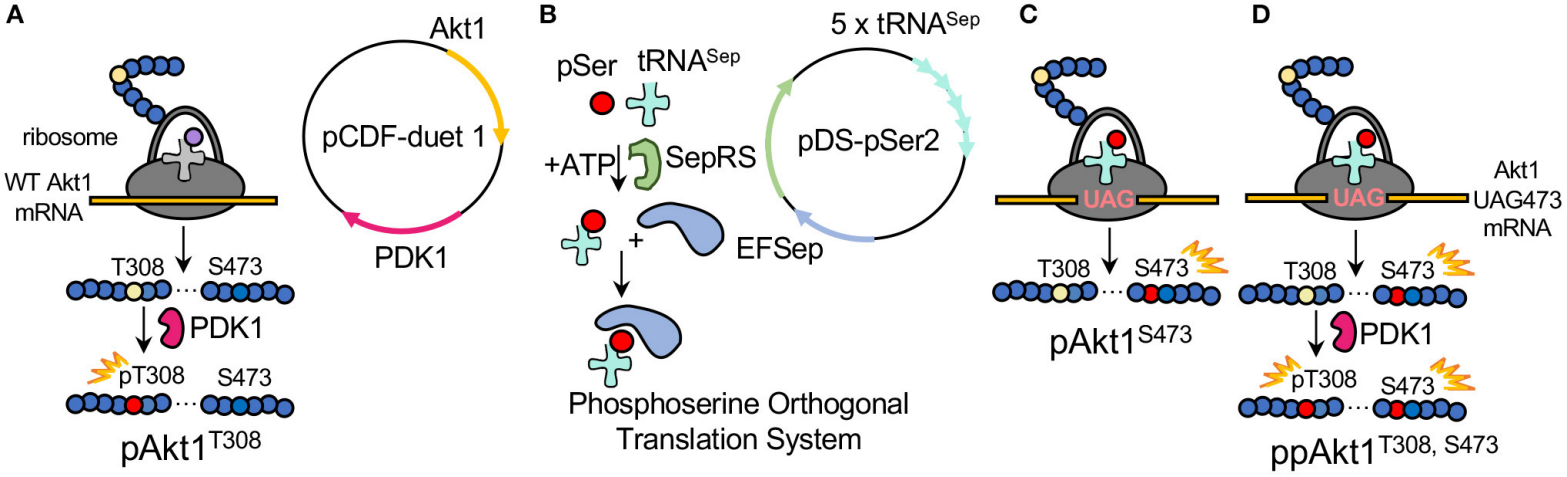
Production of differentially phosphorylated AKT1 variants. **(A)** To product pAKT1^T308^, *E. coli* was transformed with pCDF-Duet1-AKT1-PDK1 containing AKT1 and the AKT1 T308 upstream kinase PDK1. **(B)** The plasmid pDS-pSer2 contains the phosphoserine orthogonal translation system (OTS) which was used to genetically incorporate phosphoserine at position 473 in response to a UAG nonsense codon in AKT1. The OTS includes the phosphoseryl-tRNA synthetase (SepRS), an elongation factor Tu mutant (EFSep) and five copies of a tRNA^Sep^ expression cassette. The OTS is co-expressed **(C)** with a pCDF-duet1 vector containing AKT1 TAG473 and lacking the PDK1 gene. The doubly phosphorylated AKT1 is then generated by **(D)** co-expressing pDS-pSer2 with the pCDF-duet1 vector containing AKT1 TAG473 and PDK1.

Recently, we developed a protocol that allows recombinant bacterial production of AKT1 variants that are site-specifically phosphorylated at either or both regulatory sites (T308, S473) (Balasuriya et al., 2018a; Figure 3). To produce recombinant AKT1 phosphorylated at S473, an orthogonal translation system (OTS) is used to reassign the UAG stop codon to genetically encode phosphoserine (pSer). In general, an OTS requires two key components: an orthogonal aminoacyl-tRNA synthetase (o-aaRS) and cognate orthogonal transfer RNA (o-tRNA) pair. In an OTS, the aaRS charges its cognate tRNA with an unnatural amino acid (uAA) that is usually provided to *E. coli via* direct supplementation of the growth media. Orthogonality refers to the fact that the components of an OTS do not cross-react with the endogenous aaRSs and tRNAs of the expression host.

The pSer OTS used here (Figure 3B) includes a phosphoseryl-tRNA synthetase (SepRS) that was derived from an archaeal pathway for Cys-tRNA^Cys^ synthesis (Park et al., 2011), a mutant tRNA^Sep^ (Hohn et al., 2006; Aerni et al., 2015), and a mutant elongation factor-Tu (EF-Sep) (Park et al., 2011). Using the pSer OTS, we are able to reassign the UAG codon to genetically encode pSer in response to a UAG codon at position 473 in the AKT1 construct (Figures 3C,D). We previously validated this method using multiple-reaction monitoring MS/MS to unambiguously identify pSer at position 473 in AKT1 produced *via* this method with no evidence of dephosphorylation (Balasuriya et al., 2018a). In order to produce recombinant AKT1 that is phosphorylated at Thr308, the AKT1-upstream kinase PDK1 is co-expressed with AKT1. We have previously demonstrated using parallel reaction-monitoring MS/MS that co-expression of PDK1 with AKT1 results in undetectable levels of unphosphorylated Thr308 in the purified pAKT1^T308^ product (Balasuriya et al., 2018a,b).

This method of AKT1 production provided the unique opportunity to investigate the substrate specificity of individual AKT1 phosphorylated forms in a manner that was not previously possible (Balasuriya et al., 2018a, 2020). Compared to commercially available preparations of AKT1 produced using Sf9 insect cell lines that are of variable activity and contain mixtures of the active AKT1 phospho-forms (Fabbro et al., 1999; Balasuriya et al., 2018a), our protocol is able to produce consistent preparations of each individual phospho-form (Balasuriya et al., 2018a,b). Using these reagents, we defined the specific role that each phosphorylation has on AKT1 activation (Balasuriya et al., 2018a) and inhibition by clinically relevant compounds (Balasuriya et al., 2018b). We further showed that AKT1 phosphorylation status globally regulates substrate specificity (Balasuriya et al., 2020).

To study the substrate-specificities of individual AKT1 phospho-forms, our previous work tested an oriented peptide array library (OPAL) which contained library of ~10^11^ potential AKT1 substrates (Balasuriya et al., 2020). The potential substrates in the OPAL screen were partially degenerate peptides that had the following composition: Biotin-AGG-X_−6_X_−5_X_−4_R_−3_X_−2_X_−1_S_0_X_1_X_2_X_3_A; X represents any amino acid other than Ser, Thr, or Cys. The OPAL was characterized using standard radiolabeled kinase assays in separate reactions each catalyzed by a different AKT1 phospho-form. Data from the OPAL screen revealed that each phospho-form had both common and distinct preferences for specific residues at the positions surrounding the phosphorylation site. The pAKT1^S473^ optimal target motif differed significantly compared to pAKT1^T308^ and ppAKT1. The peptide library approach is a standard method used to define the consensus substrate motif for a given kinase (Obata et al., 2000; Rodriguez et al., 2004), but the approach was not previously used to differentiate the substrate specificity of distinct AKT1 phospho-forms. The results of the OPAL screens showed that each of the AKT1 phospho-forms had a distinct substrate specificity profile (Balasuriya et al., 2020), demonstrating AKT1 substrate specificity is regulated by its phosphorylation status.

By searching the known human phospho-proteome, we identified highly probable but previously unidentified AKT1 substrates. Here, we synthesized peptides representing predicted AKT1 substrates based on the OPAL data and characterized their phosphate accepting activity with each AKT1 phospho-form. The peptides were divided into three sets, with each set representing peptides that were ranked highest for each of the three phospho-forms. AKT1 activity assays showed that the majority of putative substrates displayed activity similar to or, in some cases, greater than kinase activity observed with a known substrate peptide (GSK-3β). The data showed that AKT1 phosphorylated at S473 displayed selectivity for particular substrates that was distinct from the ppAKT1 enzyme. The data further support our hypothesis that S473 phosphorylation plays a fundamental role in modulating AKT1 substrate selectivity.

## Materials and Methods

### Plasmids and Bacterial Strains

Previously, we designed two expression plasmids capable of producing differentially phosphorylated forms of recombinant human AKT1 from *E. coli*. Briefly, the first expression plasmid contained a codon-optimized, PH domain, 6xHis-tagged human *AKT1* gene (residues 109–480, 45 kDa) (Δ*PH-AKT1*), which was synthesized by ATUM (Newark, CA, USA). The AKT1 gene was subcloned into an isopropyl β-D-1-thiogalactopyranoside (IPTG)–inducible T7lac promoter–driven pCDF-Duet1 vector with CloDF13-derived CDF replicon and streptomycin/spectinomycin resistance (pCDF-Duet1-ΔPH-AKT1) (Balasuriya et al., 2018a,b). In the absence of the genetic code expansion system (see below) the pCDF-Duet1-ΔPH-AKT1 vector causes *E. coli* to express an unphosphorylated and inactive AKT1. The *PDK1* gene was cloned at the second multi-cloning site (MSC2) (*Nde*I/*Kpn*I) in pCDF-Duet1-ΔPH-AKT1 to create the second expression plasmid: pCDF-Duet1-ΔPH-AKT1-PDK1. The human *PDK1* was purchased from the Harvard PlasmidID repository service (plasmid ID: HsCD00001584; Boston, MA, USA).

In the absence of the genetic code expansion system (Figure 3), expression of the pCDF-Duet1-ΔPH-AKT1-PDK1 vector in *E. coli* leads to production of a mono-phosphorylated AKT1 (pAKT1^T308^). The genetic code expansion system for phosphoserine (pSer) is encoded on the pDS-pSer2 plasmid (George et al., 2016, 2017; Balasuriya et al., 2018a), which contains five copies of tRNA^Sep^ (Aerni et al., 2015), phosphoseryl-tRNA synthetase (SepRS9), and elongation factor-Tu mutant (EFSep21) (Lee et al., 2013).

Incorporation of pSer also required site-directed mutagenesis of the Ser473 codon to TAG in the pCDF-Duet1-ΔPH-AKT1 and pCDF-Duet1-ΔPH-AKT1-PDK1 constructs to generate the following constructs (Balasuriya et al., 2018b): pCDF-Duet1-ΔPH-AKT1-473TAG and pCDF-Duet1-ΔPH-AKT1-PDK1-473TAG which produce the mono-phosphorylated AKT1 phospho-form (pAKT1^S473^) and dual-phosphorylated (ppAKT1^T308,S473^) AKT1 phospho-forms, respectively. DNA sequencing services from the London Regional Genomics Center (London, ON, Canada) and Genewiz (Cambridge, MA, USA) were used to verify the sequences of all cloned plasmids.

### Protein Production in *E. coli*

*E. coli* strain BL21(DE3) (ThermoFisher Scientific, Waltham, MA, USA) was used to express all AKT1 protein variants. The pDS-pSer2 plasmid (George et al., 2016) was used to incorporate pSer in response to a UAG codon at position 473 of Δ*PH-AKT1* in pCDF-Duet1-ΔPH-AKT1 or pCDF-Duet1-ΔPH-AKT1-PDK1.

To produce pAKT1^T308^, pCDF-Duet1-ΔPH-AKT1-PDK1 (containing Δ*PH-AKT1* at MCS 1 and *PDK1* at MCS 2) was transformed into *E. coli* BL21(DE3) and plated on LB agar plates with 50 μg/mL streptomycin. To produce pAKT1^S473^ and ppAKT1^T308,S473^, pCDF-Duet1-ΔPH-AKT1-473TAG or pCDF-Duet1-ΔPH-AKT1-473TAG-PDK1, respectively, was co-transformed with pDS-pSer2 into *E. coli* BL21(DE3) and plated on LB (LB) agar plates with 25 μg/mL kanamycin and 50 μg/mL streptomycin (Figure 3). In all cases, a single colony was used to inoculate 50 mL of LB (with 50 μg/mL streptomycin and, if needed, 25 μg/mL kanamycin), which was grown, shaking, overnight at 37°C. From this starter culture, a 10 mL inoculum was added to 1 L of LB with antibiotics (as above) and, for pSer473-containing variants only, *O*-phospho-L-serine (Sigma Aldrich, Oakville, ON, Canada) was added to a final concentration of 2.5 mM. The cultures were grown at 37°C until OD_600_ = 0.6, at which point, for pSer473-containing variants only, 2.5 mM of additional pSer was added to the culture. Protein expression was induced by adding 300 μM of IPTG at OD_600_ = 0.8. Cultures were then incubated at 16°C for 18 h. Cells were grown and pelleted at 5,000 × g and stored at −80°C until further analysis.

### Protein Production and Purification

Nickel (Ni^2+^) affinity column chromatography was used to purify the 6xHis-tagged AKT1 variants. *E. coli* cell pellets containing recombinant AKT1 variants were resuspended in lysis buffer: 20 mM HEPES pH 7.0, 150 mM NaCl, 3 mM β-mercaptoethanol, 3 mM DTT, 10 mM imidazole, 1 mM Na_3_VO_4_, 5 mM NaF, one complete mini tablet (EDTA-free protease inhibitor mixture, Roche Applied Science, Millipore Sigma ID: 11697498001) and 1 mM phenylmethylsulfonyl fluoride were added to the cell suspension at 10 mL per g of cell pellet. Resuspended cell pellets were treated with lysozyme (1 mg/mL) for 20 min, shaking at 4°C, and subsequently lysed using a French pressure cell press (American Instrument Co. Inc.) at 1,000 psi.

Cell lysates were centrifuged at 38,000 × g for 1 h at 4°C. The supernatant was collected and filtered through a 1.2 μm filter, then mixed with 0.5 mL of Ni-nitrilotriacetic acid (Ni-NTA) resin (Thermo-Fisher Scientific). Prior to the addition of the Ni-NTA resin to the cell lysate supernatant, the resin was pre-equilibrated with 10 column volumes of lysis buffer. The Ni-NTA resin-supernatant mixture was shaken gently on a rocker at 4°C for 1 h to allow the resin to bind to the 6xHis-tagged AKT1.

The Ni-NTA resin-supernatant mixture was loaded into the Ni affinity column and washed thoroughly first with 200 mL of wash buffer A (20 mM HEPES pH 7.0, 150 mM NaCl, 3 mM β-mercaptoethanol, 3 mM Dithiothreitol, 1 mM Na_3_VO_4_, 5 mM NaF, and 15 mM imidazole), followed by 100 mL of wash buffer B (20 mM HEPES pH 7.0, 150 mM NaCl, 3 mM β-mercaptoethanol, 3 mM DTT, 1 mM Na_3_VO_4_, 5 mM NaF, and 20 mM imidazole). The 6xHis-tagged AKT1 proteins were then eluted by washing the Ni-NTA resin with 25 mL of elution buffer (20 mM HEPES pH 7.0, 150 mM NaCl, 3 mM β-mercaptoethanol, 3 mM dithiothreitol, 1 mM Na_3_VO_4_, 5 mM NaF, and 100 mM imidazole). The elution samples were then added to dialysis tubing (Sigma Aldrich, D6191) and dialyzed overnight in 2 L of imidazole-free lysis buffer to remove excess imidazole. Finally, the AKT1-containing elution samples were concentrated down to ~1 mL using Pierce Protein concentration PES centrifuge tubes with 30K MWCO (Thermo Scientific, 88529) and protein concentration of each sample was determined using a Bradford assay.

### Synthesis of Potential Peptide Substrates

The peptide substrates were generated using a standard solid phase peptide synthesis protocol outlined previously (Wei et al., 2017). Briefly, an automatic Intavis AG peptide synthesizer was used to synthesize free peptides using 9-fluorenylmethyl-oxycarbonyl (Fmoc) chemistry. TentaGel Amide Resin (Intavis catalog #32.900, Charlotte, North Carolina, USA) was first deprotected to remove the Fmoc-group on the resin, then the resin was used to couple the first amino acid during the initial synthesis cycle. In each synthesis cycle, the carboxyl group of an Fmoc-protected amino acid (FmocAA-COOH) was linked to the amine group of the previous amino acid through an amide bond. All unoccupied amine groups were then blocked (acetylated) by acetic anhydride to prevent incorrect amide bond formation in subsequent cycles. Next, the Fmoc group was removed by piperidine (de-protection) to release the free amine group for coupling to the carboxyl group of the next amino acid residue.

On completion of synthesis, the free peptide-resin was incubated in a mixture containing 95% Trifluoroacetic acid (TFA), 3% Triisopropyl silane (TIPS) and 2% H_2_O to deprotect side chains and cleave the peptide from TentaGel-resin simultaneously. The cleavage mixture containing the free peptide is then drained *via* vacuum from the tubes containing the TentaGel-resin. The cleavage mixture containing the peptide products was then washed (repeated three times) with 1 mL of pre-cooled ether followed by centrifugation at 3,000 × g for 3 min and the supernatant was removed in order to precipitate and clean the free peptide products. After allowing the peptides to dry overnight they were resuspended in MilliQ water, aliquoted and stored at −20°C. A list of the synthetic peptide sequences as well as calculated and measured masses are in Table 1.

**Table 1:** List of synthetic peptides representing predicted AKT1 substrates.

	**Gene**	**P-site**	**Protein name**	**Peptide sequence**	**MW calc (Da)**	**MW obs (Da)**	**PSSM *p*-value**
pAKT1^S473^	HRH1	S396	Histamine H1 receptor	TWKRLRSHSRQYV	1717.0	1716.0	4.6 × 10^−7^
	BRPF1	S542	Peregrin	FMQRLHSYWTLKR	1766.2	1765.0	8.2 × 10^−7^
	KIAA1109	S4592	Uncharacterized protein	TSKRALSTWGPVP	1399.7	1398.8	1.6 × 10^−6^
	TMC7	S125	Transmembrane channel-like protein 7	QWKRYSSKSWKRF	1787.2	1786.0	1.6 × 10^−6^
	RANBP3L	S77	Ran-binding protein 3-like	KRVRSSSFTFHIT	1565.9	1564.9	3.1 × 10^−6^
	ZNF256	T374	Zinc finger protein 256	THQRVHTGTRPYM	1583.7	1582.8	3.3 × 10^−6^
	SKP2	S72	S-phase kinase-associated protein 2	PRKRLKSKGSDKD	1515.0	1514.0	3.4 × 10^−6^
	LPHN3	T816	Adhesion G protein-coupled receptor L3	YSKRTMTGYWSTQ	1608.9	1607.8	3.7 × 10^−6^
	CMTM4	T208	MARVEL transmembrane 4	EIQRLDTFSYSTN	1573.8	1572.8	3.9 × 10^−6^
	PITPNM2	S668	Phosphatidylinositol transfer protein 2	PRKRSDSSTYELD	1553.8	1552.8	5.2 × 10^−6^
pAKT1^T308^	CYSLTR1	T308	Cysteinyl leukotriene receptor 1	FRKRLSTFRKHSL	1676.2	1675.1	1.8 × 10^−7^
	GRAMD1C	T515	GRAM domain containing 1C	LRRRRRTFNRTAE	1732.1	1731.1	5.5 × 10^−7^
	KCNH2	S890	Potassium channel H2	QRKRKLSFRRRTD	1747.2	1746.2	1.4 × 10^−6^
	CDCA7L	S321	Cell division cycle-associated 7-like	RRHRISSFRPVED	1654.9	1654.0	1.7 × 10^−6^
	FRYL	T1959	Protein furry homolog-like	DRRRSNTLDIMDG	1548.9	1547.8	1.8 × 10^−6^
	ASH1L	S1226	Histone-lysine N-methyltransferase	QKKRRHSFEHVSL	1651.9	1651.0	1.9 × 10^−6^
	SRRM4	S125	Serine/arginine repetitive matrix protein 4	KRRRSSSYSPSPV	1506.8	1505.9	2.6 × 10^−6^
	SCN2A	S687	Sodium channel protein type 2 alpha	RKRRSSSYHVSMD	1608.9	1607.9	2.6 × 10^−6^
	PRM2	S59	Protamine-2	YRRRHCSRRRLHR	1852.2	n.d.	3.3 × 10^−6^
	SEMA4G	S713	Semaphorin-4G	GRRRKYSLGRASR	1563.1	1561.9	3.4 × 10^−6^
ppAKT1^T308,S473^	KCNH2	S890	Potassium channel H2	QRKRKLSFRRRTD	1747.2	1729.1	7.3 × 10^−7^
	CYSLTR1	T308	Cysteinyl leukotriene receptor 1	FRKRLSTFRKHSL	1676.2	1675.1	7.3 × 10^−7^
	GRAMD1C	T515	GRAM domain containing 1C	LRRRRRTFNRTAE	1732.1	1731.0	1.1 × 10^−6^
	SASH1	S407	SAM and SH3 domain-containing	SHGRTCSFGGFDL	1383.7	1382.7	3.1 × 10^−6^
	RAB11FIP2	S277	Rab11 family-interacting protein 2	PHRRTLSFDTSKM	1575.9	1574.9	4.4 × 10^−6^
	PCNXL3	S505	Pecanex-like 3	THARVLSMDGAGG	1271.6	1270.7	5.0 × 10^−6^
	FMO2	S241	Dimethylaniline monooxygenase	FHTRFRSMLRNVL	1677.1	1676.0	5.1 × 10^−6^
	ASH1L	S1226	Histone-lysine N-methyltransferase	QKKRRHSFEHVSL	1651.9	1651.0	5.4 × 10^−6^
	BTBD11	S65	Ankyrin repeat and BTB/POZ	MHSRHNSFDTVNT	1545.6	1544.7	5.7 × 10^−6^
	TRAPPC1	S132	Trafficking protein particle complex 1	FRSRLDSYVRSLP	1596.0	1594.9	5.9 × 10^−6^
Controls	GSK3B S9A	–	Glycogen synthase kinase-3β (S9A)	GRPRTTAFAESCK	1522.7	^*^	–
	GSK3B	S9	Glycogen synthase kinase-3β	GRPRTTSFAESCK	1538.7	^*^	1.1 × 10^−4^

*Position-specific score matrix (PSSM) p-values are derived from our previously oriented peptide array analysis (Balasuriya et al., 2020). The p-values represent the probability that a higher scoring peptide may be found by chance (null hypothesis) for the indicated AKT1 phospho-from. We ranked the entire human phospho-proteome by the ability of each site to confirm to PSSMs derived from the activity of each AKT1 phospho-form. The top 10 ranked (lowest p-value) peptides were synthesized. The ~18 Da discrepancy between the theoretical (1747.2 Da) and observed (1729.1 Da) masses of the KCNH2 peptide QRKRKLSFRRRTD results from mass spectrometry ionization induced thermal decomposition (TD) (Liu et al., 2015), which results in loss of the C-terminal Asp (18 Da). Some peptides (KCNH2, ASH1L, GRAMD1C, CYSLTR1) were synthesized twice independently. Not determined (n.d.)*.

* *Control peptides were purchased from SignalChem (Victoria, BC, Canada)*.

### Mass Spectrometry Validation of Peptides

Matrix-assisted laser desorption/ionization with a time-of-flight analyzer (MALDI-TOF) mass spectrometry was used to characterize the molecular weights of the synthesized peptide substrates (1.2.4) to validate successful synthesis. Briefly, a MALDI matrix consisting of alpha-cyano-4-hydroxycinnamic acid (CHCA) was prepared as 5 mg/mL in 6 mM ammonium phosphate monobasic, 50% acetonitrile, and 0.1% trifluoroacetic acid. An aliquot of the matrix was then mixed with the synthesized peptide samples at 1:1 ratio (v/v).

**Figure 4:**
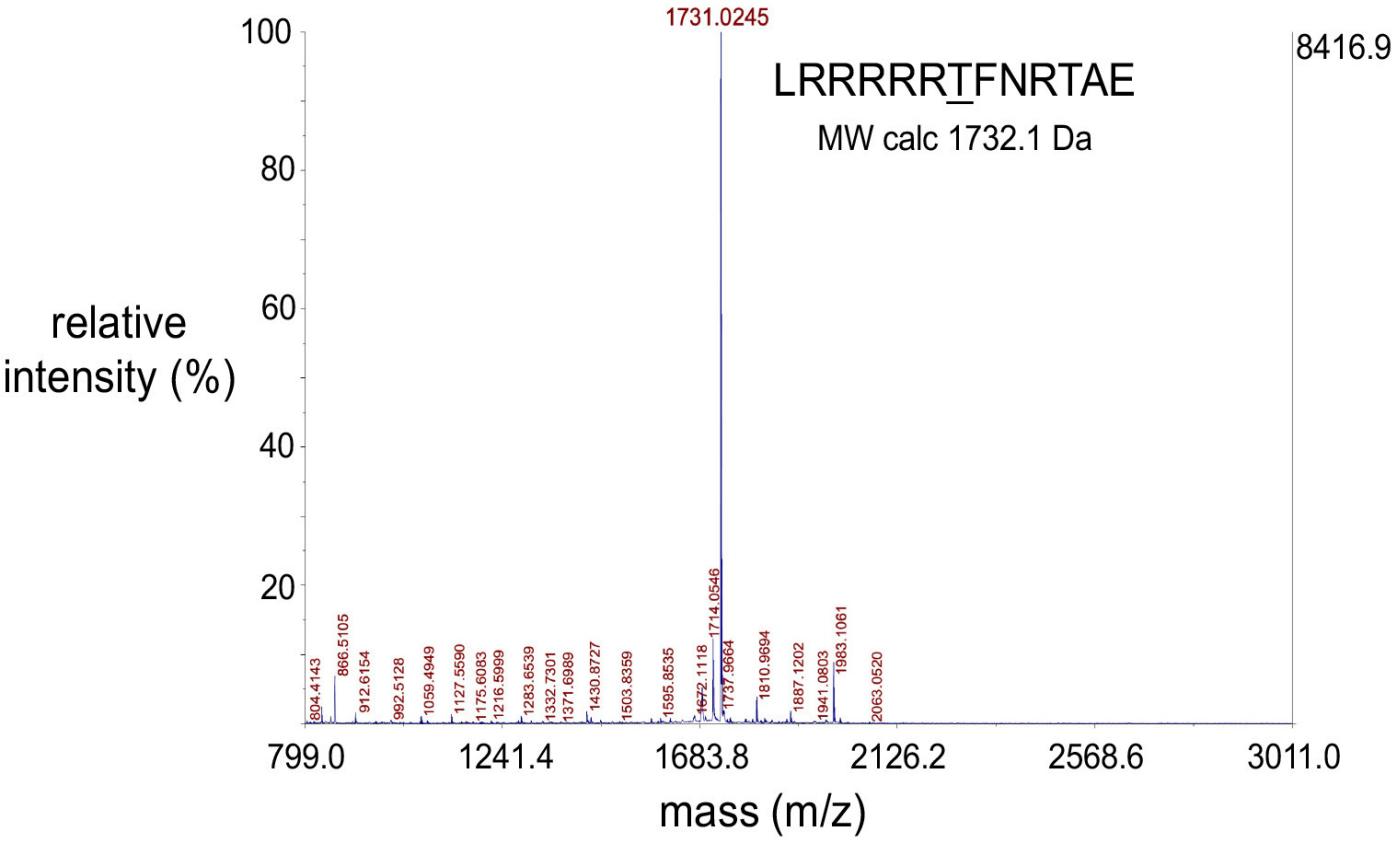
Representative MALDI-TOF spectrum of a synthesized peptide substrate. MALDI-TOF mass spectrometric data were obtained using an AB Sciex 5800 TOF/TOF System, MALDI TOF (Framingham, MA, USA). Data acquisition and data processing were, respectively, done using a TOF/TOF Series Explorer and Data Explorer (both from AB Sciex). The x-axis position of the blue peaks is measured in mass/charge number of ions (m/z). Here, the charge number of ions (z) is equal to 1 and so the (m/z) value is a measure of molecule size (m, in Daltons) which corresponds to the molecular weights of the analyzed molecules. The large peak corresponding to the molecular weight of 1731.03 Da confirms the identity of the peptide substrate as well as the integrity of the solid phase peptide synthesis protocol used to synthesize the peptides. The peptide sequence and theoretical molecular weight of the peptide is indicated. The y-axis indicates intensity in percent (left) and absolute value of ion counts (right).

MALDI-TOF mass spectrometric data were obtained using an AB Sciex 5800 TOF/TOF System, MALDI TOF (Framingham, MA, USA). Data acquisition and data processing were, respectively, done using a TOF/TOF Series Explorer and Data Explorer (AB Sciex). The instrument is equipped with a 349 nm Nd:YLF OptiBeam On-Axis laser. The laser pulse rate was 400 Hz. Reflectron positive mode was used. Reflectron mode was externally calibrated at 50 ppm mass tolerance. Each mass spectrum was collected as a sum of 1,000 shots. A representative spectrum of a single synthesized peptide substrate is shown (Figure 4) and additional MALDI-TOF spectra are in Supplementary Figure 1.

### Kinase Activity Assays

The activity of each AKT1 phospho-form was characterized by performing a quantitative *in vitro* kinase assays in the presence of 200 μM of a given potential substrate peptide (Table 1). Assays were performed in a reaction buffer consisting of 3-(N-morpholino)-propanesulfonic acid (MOPS, 25 mM, pH 7.0), 12.5 mM β-glycerolphosphate, 25 mM MgCl_2_, ethylene glycol-bis(β-aminoethyl ether)-N,N,N′,N′-tetraacetic acid (EGTA, 5 mM, pH 8.0), ethylenediaminetetraacetic acid (EDTA) (2 mM), ATP (0.02 mM), and 0.4 μCi (0.033 μM) γ-[^32^P]-ATP. Each assay (30 μL reaction volume) was performed using at least three independent enzyme reactions. The reactions were initiated with addition of enzyme and incubated at 37°C. The t = 0 time point represents a control that lacks enzyme. Time points were taken over a 5 min time course at t = 1, 2, 3, and 5 min. As previously (Balasuriya et al., 2018a,b), reactions were initiated by the addition of AKT1 (1.8 pmol of pAKT1^S473^, 0.36 pmol of pAKT1^T308^, or 0.09 pmol of ppAKT1^T308,S473^). Different concentrations of each phospho-form were used in the *in vitro* kinase assays to accommodate the varying levels of activity of each of the phospho-forms, as was done previously (Balasuriya et al., 2018a). In order to accurately determine the initial velocity (v_0_) of each reaction, enzyme concentrations were chosen such that product formation increased linearly during the 5 min time course. Activities of each Akt1 phospho-form are then compared by determining the apparent catalytic rate (k_app_ = v_0_/[Akt1]). The rate is, thus, presented below in units of fmol or pmol of phospho-peptide/min/pmol of enzyme as indicated. Reactions were performed as *N* = 3 or *N* = 6 (as indicated) independent enzyme reactions.

Aliquots of each reaction were quenched at each time point by spotting 3 μL of each reaction on P81 paper (Turowec et al., 2010). Following spotting, the 3 μL spots were first allowed to dry, then were washed with 1% phosphoric acid (3 × 10 min), then washed in 95% ethanol (1 × 5 min), and finally allowed to air-dry completely. Incorporation of γ-[^32^P] from the γ-[^32^P]-ATP into the potential substrate peptides was detected by exposing the P81 paper to a phosphor-imaging screen. The γ-[^32^P]-peptide products were imaged and quantitated using a Storm 860 Molecular Imager and ImageQuant TL software (GE Healthcare, Mississauga, ON, Canada). The phosphor-images of each reaction in Supplementary Figures 4–6.

### Statistical Analysis

Multiple statistical tests were used to identify a group of peptide substrates that were not significantly (ns) different from the background measurements determined in experiments containing only kinase enzyme and buffer but lacking substrate. The level of statistical significance between enzyme activities with each peptide compared to the group of non-significant peptides were calculated using pairwise *T*-test in Microsoft Excel.

### Sequence Logo Analysis

Sequence logos corresponding to substrate selectivity (Table 2) for each Akt1 variant were generated using WebLogo version 2.8.2 (Crooks et al., 2004). A sequence alignment was generated such that each peptide was represented with a frequency that corresponds to the selectivity values in Table 2. Thus, peptides with a high selectivity will be represented by a high entropy in bits compared to peptide substrates that show low selectivity for a particular AKT1 phospho-form.

**Table 2:** Akt1 phospho-form relative selectivity values.

**Gene**	**Protein name**	**Peptide sequence**	**pI**	**Phospho-form relative selectivity value (x-fold)**		
				**pAkt1 ^**S473**^**	**pAkt1 ^**T308**^**	**ppAkt1 ^**T308,S473**^**
ZNF256	Zinc finger protein 256	THQRVHTGTRPYM	10.83	6.7	0.3	0.3
CMTM4	MARVEL transmembrane 4	EIQRLDTFSYSTN	4.37	8.3	0.2	0.2
FMO2	Dimethylaniline monooxygenase	FHTRFRSMLRNVL	12.3	4.4	0.2	0.5
KIAA1109	Uncharacterized protein	TSKRALSTWGPVP	11	2.9	0.5	0.6
TRAPPC1	Trafficking protein particle complex 1	FRSRLDSYVRSLP	10.74	3.6	0.3	0.6
BTBD11	Ankyrin repeat and BTB/POZ	MHSRHNSFDTVNT	6.69	2.5	0.4	0.8
SCN2A	Sodium channel protein type 2 alpha	RKRRSSSYHVSMD	10.9	2.1	0.3	1.0
LPHN3	Adhesion G protein-coupled receptor L3	YSKRTMTGYWSTQ	9.7	1.4	0.6	1.0
CDCA7L	Cell division cycle-associated 7-like	RRHRISSFRPVED	11.52	1.7	0.4	1.3
HRH1	Histamine H1 receptor	TWKRLRSHSRQYV	11.72	1.0	0.6	1.5
PITPNM2	Phosphatidylinositol transfer protein 2	PRKRSDSSTYELD	6.54	0.8	0.8	1.5
KCNH2	Potassium channel H2	QRKRKLSFRRRTD	12.18	1.1	0.4	2.0
SRRM4	Serine/arginine repetitive matrix protein 4	KRRRSSSYSPSPV	11.72	0.8	0.5	2.1
CYSLTR1	Cysteinyl leukotriene receptor 1	FRKRLSTFRKHSL	12.31	0.5	0.7	2.3
RAB11FIP2	Rab11 family-interacting protein 2	PHRRTLSFDTSKM	10.84	0.6	0.7	2.3
GRAMD1C	GRAM domain containing 1C	LRRRRRTFNRTAE	12.3	0.8	0.4	2.4

*Relative selectivity values of each Akt1 phospho-form are presented for each of the peptides listed. Relative selectivity values were derived from the normalized k_app_ values of each Akt1 phospho-form for each of the peptides listed (See Results)*.

## Results

### Peptide Synthesis and Characterization

For each of the AKT1 phospho-forms, we were previously (Balasuriya et al., 2020) able to determine the amino acids that were either preferred or dis-favored according to enzyme activity at residues (−6 to +4) surrounding the substrate phosphorylation site (T/S_0_) in the consensus motif. The data were based on multiple oriented peptide libraries (OPALs) such that each site surrounding T/S_0_ was subjected to saturation mutagenesis. Because we assayed the OPALs for activity with each AKT1 phospho-form, we were able to determine how each amino acid could increase or decrease activity at each variable position in the target peptide. These AKT1 phospho-form specific OPAL data were then converted into a set of three position-specific scoring matrices (PSSMs).

Although full details are provided in Balasuriya et al. (2020), we converted the enzyme activities to PSSMs following the original method developed in ScanSite (Obenauer et al., 2003). Briefly, the enzyme activities at each site in the target motif for all possible amino acids were converted to a matrix of selectivity values after normalizing by the average activity observed over the OPAL. The PSSM is the logarithm of the selectivity values, thus, residues at positions showing above average activity contribute positively to the peptide score. Residues at positions in the target peptide associated with below average activity contribute negatively to the peptide score. The PSSMs were used to search and rank the human phospho-proteome (Balasuriya et al., 2020). The search was restricted to human phospho-sites that have been experimentally identified. The search tests each possible peptide and scores the residues at each site according to the preferences in the PSSM. For example, pAKT1^T308^ and ppAKT1 showed a strong preference for basic residues at the −5 position (Balasuriya et al., 2020), so any matching peptides will score relatively higher in the search result. Conversely, we found no such preference in the pAKT1^S473^ enzyme at −5, which led to different list of top hits for a PSSM based on this enzyme. Using the PSSM-ranked list of peptides, we then predicted the expectation that a given phospho-peptide sequence was a likely substrate for one or more of the AKT1 phospho-forms.

In order to validate substrates predicted by the OPAL data, peptide substrates were synthesized (Table 1) that represent novel putative AKT1 target peptides. The targeted peptides library included those ranked highest (top 1%; *p* < 10^−6^) in our OPAL-derived PSSM search of the human phospho-proteome (Balasuriya et al., 2020). Database searches using a separate PSSM developed for each AKT1 phospho-form (pAKT1^S473^, pAKT1^T308^, ppAKT1^S473,T308^) were used to rank and select 10 putative substrate peptides for each phospho-form (Table 1). We synthetized a total of 26 peptides as some of the top peptides where shared by pAKT1^T308^ and ppAKT1. Each phosphorylated peptide motif in the known human phospho-proteome (Hornbeck et al., 2004) was ranked based on three separate PSSM scoring systems, which predicted the likelihood that a given motif the in database was a substrate of the corresponding AKT1 phospho-form. We confined our target library to peptides that have been identified to be phosphorylated yet the upstream is not known or not known to include AKT1.

The peptide substrates were synthesized using solid state peptide synthesis (see Methods). Quality of the synthetic peptides was determined using MALDI-TOF mass spectrometry. MALDI-TOF spectra verified purity and identity of the peptides (Figure 4, Supplementary Figure 1, Table 1). The spectra positively identified expected peptides by the presence of a sole major peak at the expected molecular weight of the peptide. Additional MALDI-TOF spectra confirmed synthesis of pure and accurate peptides (Supplementary Figure 1). For one peptide, derived from KCNH2 [QRKRKLSFRRRTD], there was a discrepancy of 18 Da between the theoretical and observed masses of the synthesized peptide due to ionization induced thermal decomposition (TD) (Liu et al., 2015). TD can cause peptide dehydration (mass – 18 Da) in cases where the peptide contains a C-terminal aspartic acid.

### Benchmarking Activity of the AKT1 Phospho-Forms

Each of the AKT1 phospho-forms were purified using affinity chromatography as before; see (Supplementary Figure 2) and references (Balasuriya et al., 2018a,b, 2020). Previous investigations from our lab have determined that the deletion of the PH domain from AKT1 abolishes its auto-inhibitory activity and improves the purification of AKT1 by increasing its solubility (Balasuriya et al., 2018b, 2020). Thus, the AKT1 phospho-forms used in this present study (AKT1 residues 109–472) lacked a PH domain (Supplementary Figure 2). We previously characterized each AKT1 phospho-form biochemically and with mass spectrometry to confirm site-specific phosphorylation for each AKT1 variant produced in this manner (Balasuriya et al., 2018a,b).

Activity levels for all three AKT1 phospho-forms (ppAKT1 ≥ pAKT1^T308^ >>> pAKT1^S473^) prepared in this study were consistent with previously prepared AKT1 phospho-forms (Balasuriya et al., 2018a,b; Supplementary Figure 3). Our previous characterization over a large defined peptide library showed that ppAKT1 is more active (by ~1.5–3-fold) than pAKT1^T308^ on ~50% of peptides representing known AKT1 substates, while the two enzymes have similar activity on most of the remaining peptides and a few peptides showed greater activity with pAKT1^T308^ (Balasuriya et al., 2020). Both enzymes phosphorylated at T308, however, display ~100-fold greater activity than the enzyme phosphorylated only at S473. Furthermore, in COS7 cells, we found that phosphorylation at T308 was necessary and sufficient for maximal AKT1 signaling, while enzyme phosphorylated only at S473 could not achieve measurable signaling activity using a fluorescent AKT1 activity reporter (BKAR) (Balasuriya et al., 2018a). The primary role of T308 in activating AKT1 is also evident in its central position in the AKT1 active and peptide binding sites. The S473 site is located in a regulatory loop, called the hydrophobic motif, that is distant form the active site (Figure 2).

In order to verify the activity of the purified AKT1 phospho-forms produced in this study, *in vitro* kinase assays were conducted using the known AKT1 substrate GSK-3β as well as a negative control peptide in which the S_0_ residue is mutated to alanine (A_0_) (Supplementary Figure 6). To confirm consistent activity between the AKT1 preparations, activity values from previously purified and experimentally validated (Balasuriya et al., 2018b) AKT1 phospho-forms were compared to the new preparation used here (Supplementary Figure 3). These data demonstrate the consistency of the enzyme activity that is achievable by our method.

### Activity of Predicted AKT1 Substrate Peptides

The predicted AKT1 peptide substrates were then subjected to kinase assays. The activity data are based on a reaction time course during which the transfer of radiolabeled phosphate to the substrate peptide is quantitated (Supplementary Figures 4, 5). The initial velocities of the enzymatic reaction (*v*_0_) were determined by linear regression of the time courses. The initial velocity data (Supplementary Figure 7) were used to compare AKT1 activity over different peptide substrates including a known AKT1 substrate GSK-3β positive and GSK-3β (A_0_) negative controls (Supplementary Figure 6).

The predicted peptide substrates were assayed using the same AKT1 phospho-form that was used to develop the PSSM scoring system that predicted the given peptide (Table 1). From these assays, the three highest and three lowest scoring peptides for each variant were selected for additional kinase assays conducted with the remaining two AKT1 phospho-forms. For example, from the initial assays of the 10 peptides predicted to be substrates of pAKT1^S473^ (Table 1), peptides representing high (CMTM4, LPHN3, and PITPNM2) and low (KIAA1109, HRH1, and ZNF256) activity were subsequently assayed with the remaining two AKT1 phospho-forms: pAKT1^T308^ and pAKT1^T308,S473^. In total, 16 of the 26 total peptides were assayed using all three AKT1 phospho-forms (*v*_0_) (Supplementary Figure 7).

**Figure 5:**
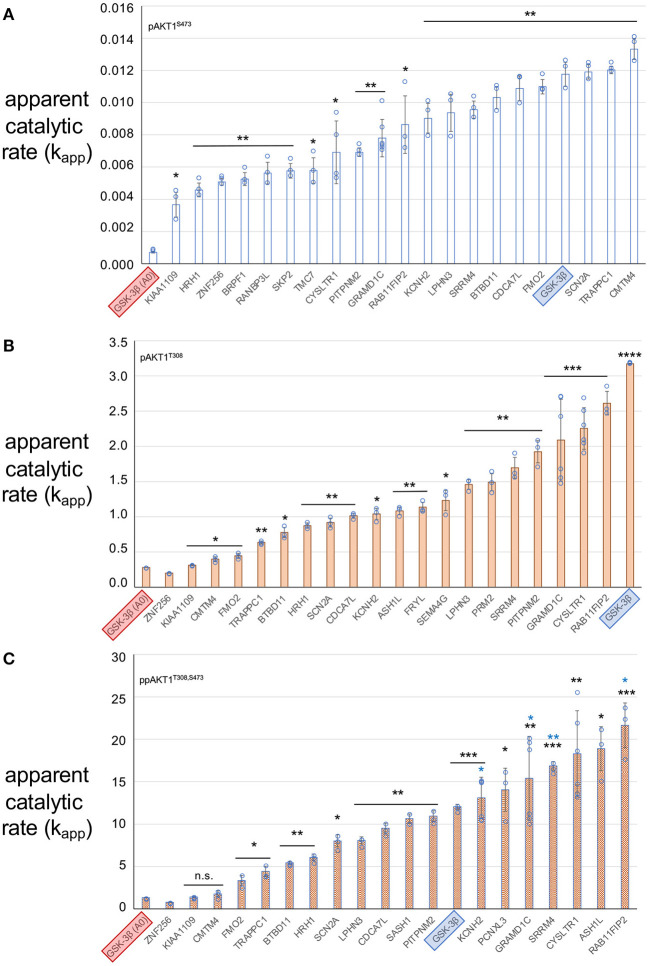
Apparent catalytic rate (k_app_ – pmol of phospho-peptide/min/pmol of enzyme) of each AKT1 phospho-forms with peptide substrates. **(A)** pAKT1^S473^, **(B)** pAKT1^T308^, and **(C)** ppAKT1^T308,S473^. Each bar represents the average k_app_ value of 3 replicates and the value of each replicate is indicated by a closed circle. All three AKT1 phospho-forms were tested using the known AKT1 substrate GSK-3β (SGRPRTT*S*FAESCKP, blue highlight) as a standard to assess the activity of the AKT1 preparations, as well as a negative control variant of GSK-3β: GSK-3β A_0_ (SGRPRTT*A*FAESCKP, red highlight). GSK-3β A_0_ contains an un-phosphorylatable alanine residue in place of the serine residue that is normally phosphorylated by AKT1. Error bars show two standard deviations about mean for at least three independent reactions (N = 3 or N = 6 independent enzyme reactions). The level of statistical significance compared to GSK-3β A_0_ are indicated with black asterisks (*p < 0.05; **p < 0.005; ***p < 0.0005; ****p < 0.00005; n.s., not significant). Blue asterisks indicate significantly greater activity than the positive control GSK-3β peptide (*p < 0.05; **p < 0.005).

Different concentrations of each AKT1 phospho-form were used in the kinase assays (see Methods) to permit accurate calculation of *v*_0_. Because initial velocity is computed from the linear phase of the enzyme reaction, conditions must be used to ensure the reactions proceeds linearly during the time course. Adjusting the level of enzyme enables accurate determination of *v*_0_. To account for these differences in protein concentration when comparing activity levels across the AKT1 phospho-forms, *v*_0_ values were divided by the AKT1 enzyme concentration used in the given assay to arrive at the apparent catalytic rate (*k*_*app*_) of each reaction (Figure 5). The *k*_*app*_ values obtained here agree with the previously observed trend in AKT1 activity (Balasuriya et al., 2018a,b), in which the pAKT1^S473^ enzyme shows significant activity above the negative control, across all substrates tested, that is far below the activity level for the pAKT1^T308^ and ppAKT1^T308,S473^ enzymes. We also observed that ppAKT1^T308,S473^ is more active than pAKT1^T308^ on the GSK-3β peptide, as we noted before (Balasuriya et al., 2018a,b; Balasuriya et al., 2020; Figure 5).

We found that all substrates showed significant phosphate accepting activity with at least one of the AKT1 phospho-forms. Indeed, some of the putative substrates displayed greater activity than well-established AKT1 substrate peptide. ppAKT1^T308,S473^ was significantly more active (*p* = 0.0053) with the peptide derived from RAB11FIP2 than with the known AKT1 substrate GSK-3β. Pairwise comparisons of *k*_*app*_ values for each peptide tested between pAKT1^T308^ and ppAKT1^T308,S473^ revealed that ppAKT1^T308,S473^ was at least as active as pAKT1^T308^ with 6 (33%) of the 18 peptides tested. In agreement with our previous observations (Balasuriya et al., 2020), a substantial portion of the substrates show same activity regardless of S473 phosphorylation. Peptides representing CMTM4, FMO2, TRAPPC1, BTBD11, and KIAA1109 showed no significant difference in *k*_*app*_ for ppAKT1 compared to pAKT1^T308^. The doubly phosphorylated enzyme was more active (*p* < 0.009) with 12 (66%) of the peptides. Interestingly, the six peptides that had similar *k*_*app*_ values for both pAKT1^T308^ and ppAKT1^T308,S473^ were also the six least active peptides for both phospho-forms. The data again re-affirm our observations that rather than simply increasing activity, S473 phosphorylation modulates AKT1 activity in a substrate dependent fashion.

### Substrate Selectivity in AKT1 Variants Phosphorylated at S473

**Figure 6:**
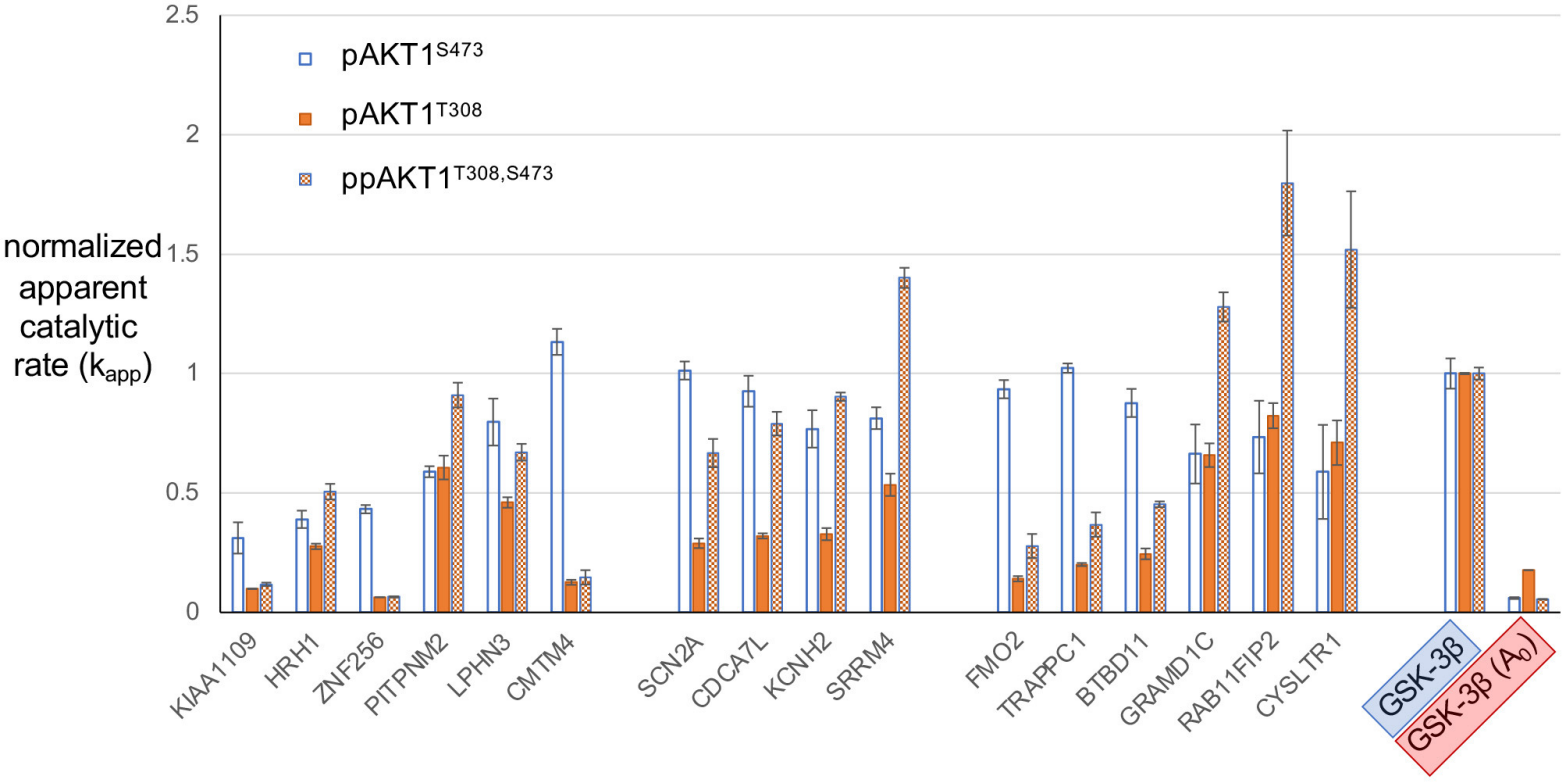
Normalized apparent catalytic rate (k_app_) of the phosphorylation reaction catalyzed by different AKT1 phospho-forms on predicted AKT1 substrate peptides. The k_app_ values of each phospho-form (pAKT1^S473^ (blue, open bars), pAKT1^T308^ (orange, solid), and ppAKT1^T308,S473^ (orange, checkered) have been normalized such that the average activity of each AKT1 variant with GSK-3β is set to 1.0. Each of the peptides shown here were individually subjected to *in vitro* kinase assays with each of the three AKT1 phospho-forms. Error bars represent two standard deviations about mean for three independent enzyme reactions (*n* = 3).

In order to estimate the relative preference or selectivity of each AKT1 phospho-form for the series of peptides, normalized *k*_*app*_ values for each of the peptides tested were plotted (Figure 6). This analysis allows us to identify peptides and features of peptide substrates that are most favored by each of the AKT1 phospho-forms. Normalization was based on setting the *k*_*app*_ for each AKT1 variant for the positive control GSK-3β peptide to 1.0. The normalized apparent rates revealed trends in the substrate selectivity of pAKT1^S473^ compared to the pAKT1^T308^ and ppAKT1^T308,S473^. To quantify the relative selectivity of each phospho-form for each peptide, we calculated relative selectivity values (Table 2). The phospho-form selectivity values were estimated by dividing one AKT1 phospho-form's *k*_*app*_ value for a given peptide by the average of the remaining two phospho-form's *k*_*app*_ values for the same peptide.

Our goal here was to identify peptides that are most favored by one of the AKT1 phospho-forms but not by the others. For example, to arrive at a selectivity value for pAKT1^S473^ with ZNF256, pAKT1^S473^ normalized *k*_*app*_ for ZNF256 (0.43) was divided by the average of normalized rates for pAKT1^T308^ and pAKT1^T308,S473^ with ZNF256 (0.06) to arrive at a selectivity value for pAKT1^S473^ with ZNF256 of 6.7-fold. A selectivity value of 1 indicates that the phospho-form is relatively as active on the given peptide as the other two phospho-forms, where as a selectivity value above or below 1 indicates that the given phospho-form is relatively more or less active on the given peptide than the other two phospho-forms, respectively. From these values (Table 2), it is apparent that the pAKT1^S473^ phospho-form has a strong selectivity (>2-fold) toward the peptide substrates derived from ZNF256, KIAA1109, CMTM4, FMO2, TRAPPC1, and BTBD11. Moreover, the ppAKT1^T308,S473^ phospho-form appears to have a strong selectivity (>2-fold) toward a completely distinct set of peptide substrates derived from GRAMD1C, SRRM4, CYSLTR1, and RAB11FIP2. The pAKT1^T308^ enzyme showed robust activity with most peptides, but this enzyme was not particularly selective for any of the substrates when compared with pAKT1^S473^ and the doubly phosphorylated enzyme. Taken together these results support the idea that AKT1 phosphorylation status, and S473 phosphorylation specifically, plays an important role on determining AKT1 substrate specificity.

### Analysis of Residue Preferences in the Substrate Peptides

**Figure 7:**
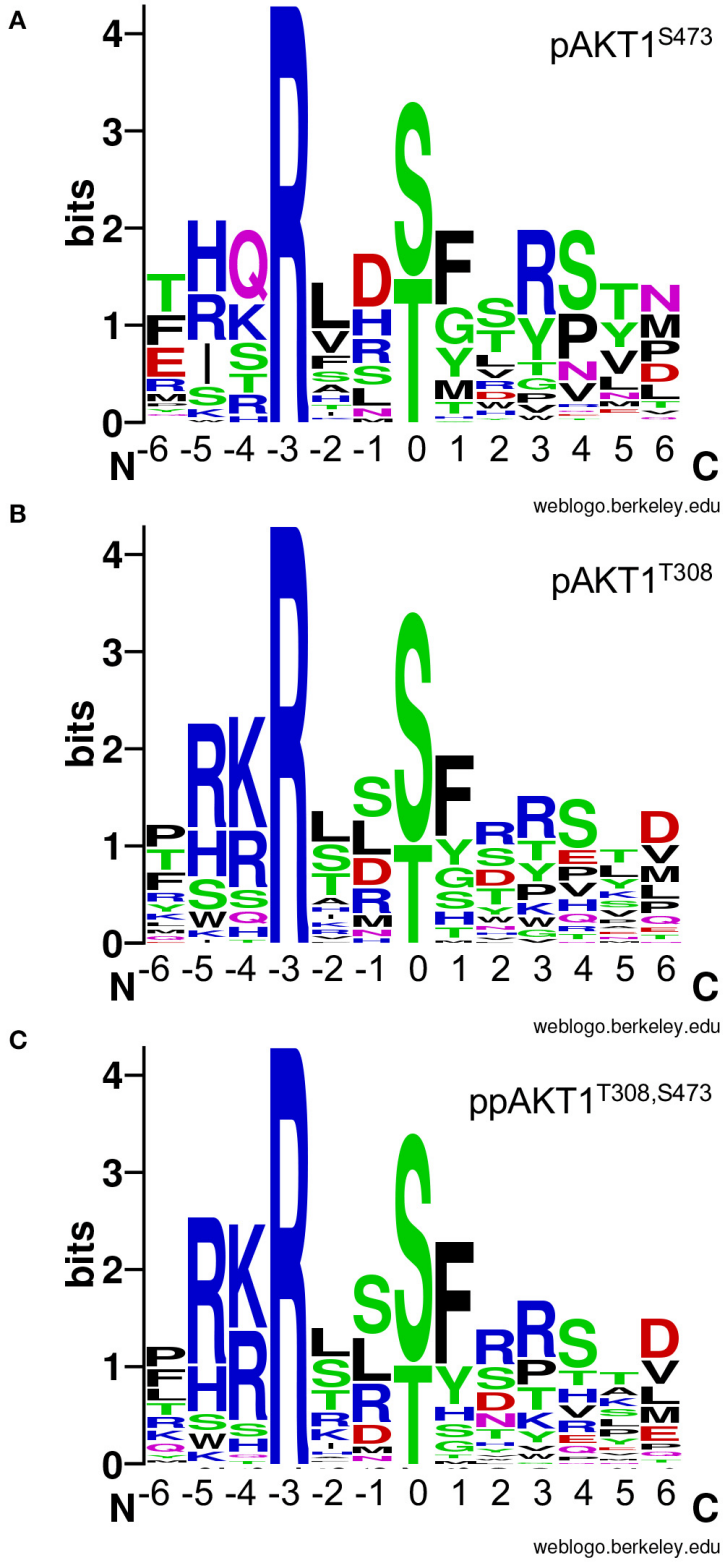
AKT1 phospho-form selectivity-based sequence logos. Sequence logos for **(A)** pAKT1^S473^, **(B)** pAKT1^T308^, and **(C)** ppAKT1^T308,S473^ are based on the enzyme selectivity values (Table 2) for each peptide in our targeted library of putative AKT1 substrates. The entropy values are linearly related to enzyme selectivity (see Table 2, Methods). The phosphorylation site is denoted by the 0 position. Sequence logos were created using the WebLogo server (Crooks et al., 2004).

We then used the selectivity values to generate sequence logos that represent each sequence in relation to the selectivity value for the given peptide (Figure 7). This has the effect of providing residues in highly selective peptide with the highest entropy values in the sequence logo. Low entropy values represent sequences that were not favored by a given AKT1 phospho-form. The logos show greater similarity in the residue preference for the two enzymes phosphorylated at T308 compared to pAKT1^S473^.

The pAKT1^S473^ enzyme shows no preferences for the nature of the residue at position −5. This is an interesting finding, because R_−5_ is normally regarded as an important part of the AKT1 consensus (Obata et al., 2000). The pAKT1^S473^ enzyme shows equal selectivity for R, H, I, or S at this site (Figure 7A). This is in contrast to the pAKT1^T308^ and ppAKT1 enzymes that strongly prefer R_−5_ (Figures 7B,C). This result is in accord with our larger OPAL data set, which showed that the pAKT1^S473^ enzyme did not show a strong preference for any particular amino acid at −5, while the pAKT1^T308^ and ppAKT1 enzymes strongly preferred basic amino acids (R, H, and K) (Balasuriya et al., 2020). Indeed, ppAKT1 shows the strongest selectivity for substrates with basic residues at −5 and −4. Similarly, while all enzymes show a strong preference for an aromatic residue at +1, this preference is greatest for the ppAKT1 enzyme. Finally, we observed a greater preference for Ser+4 and Pro+4 with the pAKT1^S473^ enzyme, and this enzyme favored more acidic peptides, e.g., Glu_−6_ and Asp_−1_ (Figure 7). The analysis suggests that the pS473 and ppAKT1 enzymes generate selectivity by recognizing different regions of the substrate motif. The ppAKT1 enzyme is more selective for particular residue types at −5, −4, and +1 while the pS473 enzyme is more tolerant of sequence diversity at these sites.

## Discussion

### Comparing the Genetic Code Expansion System to Alternative Methods of Producing Activate AKT1

We use genetic code expansion in an *E. coli* expression system to incorporate phosphoserine into AKT1 at position 473. When co-expressed with the AKT1 upstream kinase PDK1, our approach allows production of site specifically phosphorylated variants of AKT1 that are otherwise difficult to isolate. Prior to the development of the genetic code expansion system used here, initial attempts at producing recombinant active AKT1 employed the use of phospho-mimetic mutations (Klein et al., 2005). Phospho-mimetic mutations involve replacing the serine at position 473 with glutamate as its side chain was proposed to mimic the structure and function of a phosphorylated serine residue. Earlier studies were unable to compare the activity of phospho-mimetic S473E AKT1 construct to AKT1 that contained only pSer at position 473 (Klein et al., 2005). We successfully produced recombinant AKT1 with pSer at position 473 and found that phospho-mimetic substitutions do not accurately mimic the functional role of phosphorylation in activating AKT1 (Balasuriya et al., 2018a). Indeed, we found the use of phospho-mimetics were unable to activate the enzyme, while phospho-ablated mutants (alanine substitutions at T308 and S473) actually inactivated the enzyme. These findings call into question the validity of phospho-mimetics as a replacement for phosphorylation and also demonstrate the applicability of the AKT1 constructs produced by the genetic code expansion approached.

### Validating Predicted AKT1 Substrates

Using our established method (Balasuriya et al., 2018a,b, 2020; Chung et al., 2019) to produce site-specifically phosphorylated variants of AKT1, we recently conducted *in vitro* kinase assays of OPALs that contained degenerate peptides representating many putative AKT1 substrates (Balasuriya et al., 2020). By measuring the activity of each separate AKT1 phospho-form (pAKT1^S473^, pAKT1^T308^, ppAKT1^T308,S473^) using the OPAL, we were able to detect differences in substrate specificities for each of the AKT1 phospho-forms. Based on the activity data we had measured for all possible amino acids at each site in the substrate motif, we developed a PSSM scoring system that we then used to evaluate and predict potential substrates for each of the AKT1 phospho-forms. Previously, the inability to produce AKT1 in site-specifically phosphorylated forms had precluded the study of the effects of individual phosphorylation events on substrate specificity. The data enabled us to predict novel AKT1 substrates for the purpose of better characterizing the scope of substrates that are targeted by each AKT1 phospho-form.

Here, we synthesized peptides representing the “top 10” most likely putative AKT1 substrates for each phospho-form. Overall, the results presented here are congruent with the findings of our initial investigations into phosphorylation-status dependent AKT1 activity (Balasuriya et al., 2018a,b) and substrate specificity (Balasuriya et al., 2020). In particular, the general trend of AKT1 activity observed here: pAKT1^S473^ < < pAKT1^T308^ < ppAKT1^T308,S473^, mirrors the overall trend we and others have observed (Alessi et al., 1996; Balasuriya et al., 2018a,b, 2020). We did again observe, however, that AKT1 activity is dependent on the substrate. Several substrates showed equal activity with pAKT1^T308^ and ppAKT1.

AKT1 was shown to phosphorylate peptides derived from previously uncharacterized substrates *in vitro* and many of the novel peptide substrates were phosphorylated to a degree comparable to the well-established AKT1 substrate GSK-3β. The use of peptides derived from potential substrates represents a critical first step toward the validation of novel AKT1-substrates. Indeed, the results presented here validate the peptide array approach in predicting novel AKT1 substrates. The use of peptide substrates alone is suggestive but not complete evidence of a true interaction between AKT1 and the protein from which the peptide is derived. For example, conformational limitations may exist within the fully intact protein that preclude AKT1 phosphorylation, however, all of the sites considered here are known to be phosphorylated in human cells and many phosphorylation sites occur in loops or linear motifs that can be accurately mimicked by peptides. As we noted before (Balasuriya et al., 2018a), scaffolding, substrate expression or localization may also all impact the ability of AKT1 to target a particular substrate in the cell. The ability of AKT1 to efficiently phosphorylate a substrate peptide is a strong evidence in favor of an AKT1-substrate relationship, yet full validation of novel AKT1-substrate relationships will require further experimentation in cells. Our data suggest that the most active peptides are strong candidates as new AKT1 substrates.

### Discovering Novel AKT1 phospho-Form-Specific Substrates

The selectivity values in Table 2 indicate novel AKT1 phospho-form-specific substrates characterized in this study. Interestingly, none of substrates showed strong selectivity for the pAKT1^T308^ enzyme compared to the other two phospho-forms. Here we characterized a total of 26 putative AKT1 substrates. For all synthetic peptides tested, significant activity above the negative control was detected (*p* < 0.0001 in all cases). Of the peptides, seven were relatively selective (Table 2 selectivity values >2) for pAKT1^S473^ (ZNF256, KIAA1109, CMTM4, FMO2, TRAPPC1, BTBD11, SCN2A) and four were relatively more selective for ppAKT1^T308,S473^ (GRAMD1C, SRRM4, CYSLTR1, RAB11FIP2). Here we defined relative selectivity to indicate that the given substrate was relatively highly active for the indicated AKT1 phospho-form and showed low relative activity for the remaining two phospho-forms.

Our analysis of the putative AKT1 substrates showed that each peptide confirmed generally to the established consensus motif R_−5_X_−4_R_−3_X_−2_X_−1_S/T_0_φ_1_ and also showed phospho-form specific differences as we observed before (Balasuriya et al., 2020). The substrates that lacked an Arg residue at the −5 position were strongly disfavored by both pAKT1^T308^ and pAKT1^T308,S473^ (selectivity values <1, Table 2) but were relatively more favored by pAKT1^S473^ (selectivity values >1, Table 2). The same pattern was observed in all seven of the novel peptides that were pAKT1^S473^-selective substrates (**Figure 6;** Table 2). Accordingly, the four novel peptides that were validated as being ppAKT1^T308,473^-selective substrates all contained an Arg/Lys residue at positions −5 and −4, and a F/Y at +1, which is likewise in agreement with our findings (Balasuriya et al., 2020).

### pAKT1^S473^-Selective Substrates

Strikingly, the pAKT1^S473^ enzyme showed no preference for basic residues at the −5 or −4 position. Because R_−5_ is normally regarded as a consensus residue for AKT1 substrates (Obata et al., 2000). Our data suggest, pAKT1^S473^ has substrate preferences that are distinct from the enzymes phosphorylated at T308. Because the consensus motif was original generated based on studies of the enzyme phosphorylated at T308, this motif will therefore fail to identify highly specific pAKT1^S473^ substrates. The pAKT1^S473^ variant also preferred Ser_+4_ or Pro_+4._ The peptide derived from the MARVEL transmembrane 4 (CMTM4) protein had the highest selectivity value for pAKT1^S473^. Accordingly, the CMTM4 peptide also exhibited the highest *k*_*app*_ of all the peptides tested with pAKT1^S473^, similar to the *k*_*app*_ for GSK-3β. Interestingly, the peptide derived from CMTM4 had the lowest isoelectric point of all the peptides tested, perhaps indicating a preference or higher tolerance for negatively charged substrates by pAKT1^S473^. Indeed, sequence logo analysis suggested a strong preference for Asp at −1 (Figure 7A). CMTM4 is a ubiquitously expressed transmembrane protein that has recently gained attention as a potential contributor to various cancers (Li et al., 2015; Bei et al., 2017; Mezzadra et al., 2017; Chrifi et al., 2019; Xue et al., 2019). As of yet, there is minimal information on the relationship between AKT1 and CMTM4 activity, and none on the role of phosphorylation at the CMTM4 T208 site that was the source of the peptide used in this study. Topology predictions using the transmembrane topology prediction software Phobius (Kall et al., 2004) suggest that the T208 site is located within the cell cytosol and is thus would be accessible for phosphorylation by AKT1.

The function of CMTM4 with respect to its role in oncogenic phenotypes appears to be highly context dependant. In clear cell renal cell carcinomas, restoration of CMTM4 expression induces G2/M cell cycle arrest (Li et al., 2015). In patients with hepatocellular carcinomas, the expression of CMTM4 was reduced compared to matched adjacent non-tumor tissues, and this reduced expression was associated with decreased overall survival rates (Bei et al., 2017). In colorectal adenocarcinomas cell lines, over-expression of CMTM4 (in the CMTM4- SW480 cell line) was associated with impeded cell proliferation, reduced migration and reduced levels of AKT phosphorylation (Xue et al., 2019). In SW480 cells, CMTM4 expression was found to be inversely related to the phosphorylation of AKT1, and the tumor-suppressive functionalities observed with CMTM4 overexpression could be mimicked in the CMTM4- SW480 cell line by direct chemical inhibition of AKT using LY294002 (Xue et al., 2019).

### ppAKT1^T308,S473^-Selective Substrates

The ppAKT1^S473,T308^ enzyme was highly selective for peptides containing a patch of basic residues at −5 and −4 and an aromatic residue at +1. Arg_−3_ is required for AKT1 activity (Obata et al., 2000; Balasuriya et al., 2020). This observation is in agreement with the peptide library and array data that we published previously (Balasuriya et al., 2020). The peptide derived from CYSLTR1 was among the most selective for ppAKT1^T308,S473^. CYSLTR1 is a cell surface receptor of the G-protein coupled receptor family that recognizes a family of inflammatory lipid mediators called cysteinyl leukotrienes (Peters-Golden et al., 2006). CYSLTR1 is a multifunctional mediator of allergic rhinitis (Peters-Golden et al., 2006; Tsai et al., 2016). CYSLTR1 immunomodulation in allergic rhinitis was determined to involve a concurrent increase in the level of active, phosphorylated ERK1/2. Phosphorylated ERK1/2 is involved in cell adhesion, proliferation, differentiation, and cell cycle progression (Figueroa et al., 2001) and the inhibition of ERK1/2 has been shown to reduce allergic inflammation (El-Hashim et al., 2012). CYSLTR1 is not known to be an AKT1 target, nor have any direct interactions between CYSLTR1 and AKT1 been noted in the literature. However, cross-talk between the AKT1/PI3K and ERK1/2 signaling pathways has been previously implicated to be involved in both arteriogenesis (Ren et al., 2010), as well as cell motility and invasion (Gentilini et al., 2007). Thus, the link between AKT1 and CYSLTR1 may be direct *via* AKT1-mediated phosphorylation of CYSLTR1 T308 residue (the motif represented here) or indirectly through the CysLTR1-ERK1/2-AKT1 axis.

Of all the peptides tested with ppAKT1^T308,S473^, the peptide derived from RAB11FIP2 had the highest *k*_*app*_ and was one of three peptides (Figure 5) to achieve a *k*_*app*_ that was significantly higher than the known AKT1 substrate GSK-3β. RAB11FIP2 function involves the regulation of endosomal vesicle recycling between the endosomal recycling compartment and the plasma membrane (Lindsay and McCaffrey, 2004). RAB11FIP2 is not known to be a direct target of AKT1, though independent studies have posited functional (Xu et al., 2016) as well as spaciotemporal (Lindsay and McCaffrey, 2004) links between the two proteins. A recent study investigating the relationship between RAB11FIP2 expression and colorectal cancer progression demonstrated that the AKT1/PI3K pathway was involved in the RAB11FIP2-mediated expression of the oncogenic Matrix metalloproteinase 7 (MMP7) (Xu et al., 2016). An investigation on subcellular localization demonstrated that RAB11FIP2 colocalizes and indeed interacts with PIP3 in a manner similar to AKT1 (Lindsay and McCaffrey, 2004). Like the AKT1 PH domain, RAB11FIP2 contains a domain which promotes association with PIP3 at the cellular membrane. Thus, AKT1 and RAB11FIP2 may co-localize in response to the same cellular stimuli that converts PIP2 to PIP3.

## Data Availability Statement

The original contributions presented in the study are included in the article/Supplementary Material, further inquiries can be directed to the corresponding author.

## Author Contributions

MM and PO'D analyzed data and wrote the paper. MM, NB, and PO'D edited the paper. MM and SZ performed experiments. MM, NB, SZ, SL, and PO'D designed experiments. PO'D and SL provided funding and supervised the work. All authors contributed to the article and approved the submitted version.

## Conflict of Interest

The authors declare that the research was conducted in the absence of any commercial or financial relationships that could be construed as a potential conflict of interest.
