# Supplementary material for: Phospho-Form Specific Substrates of Protein Kinase B (AKT1)
Source: Front Bioeng Biotechnol. 2021 Feb 3;8:619252. doi: 10.3389/fbioe.2020.619252 (PMC7886700; doi:10.3389/fbioe.2020.619252)
Supplement: Supplementary file 1 [file Data_Sheet_1.DOCX]

Supplementary Material for:

Phospho-form specific substrates of protein kinase B (AKT1)

McShane McKenna^1^, Nileeka Balasuriya^1^, Shanshan Zhong^1^, Shawn Shun-Cheng Li^1^, and

Patrick O’Donoghue^1,2,*^

^1^Department of Biochemistry, ^2^Department of Chemistry, The University of Western Ontario, London, Ontario N6A 5C1, Canada.

*** Correspondence:**Corresponding Author
[patrick.odonoghue@uwo.ca](mailto:patrick.odonoghue@uwo.ca)

# Supplementary Data

The supplementary material contains Supplementary Figures S1-S7 and Supplementary References.

# Supplementary Figures and Tables

## Supplementary Figures

TWKRLRSHSRQYV

MW calc 1717.0 Da

Figure S1. MALDI-TOF spectrum of a synthesized peptide substrate. MALDI-TOF mass spectrometric data were obtained using an AB Sciex 5800 TOF/TOF System, MALDI TOF (Framingham, MA, USA).  Data acquisition and data processing were respectively done using a TOF/TOF Series Explorer and Data Explorer (both from AB Sciex). The x-axis scale is equivalent to mass in Daltons (Da) as the charge number of ions (z) is equal to 1. The large peak (A) corresponding to the molecular weight of 1731.03 Da confirms the identity of the peptide substrate as well as the integrity of the solid phase peptide synthesis protocol used to synthesize the peptides. The y-axis indicates intensity in percent (left) and absolute value of ion counts (right).

TSKRALSTWGPVP

MW calc 1399.7 Da

QWKRYSSKSWKRF

MW calc 1787.2 Da

**Figure S1** continued.

PRKRLKSKGSDKD

MW calc 1515.0 Da

FMQRLHSYWTLKR

MW calc 1766.2 Da

**Figure S1** continued.

FRKRLSTFRKHSL

MW calc 1676.2 Da

LRRRRRTFNRTAE

MW calc 1732.1 Da

**Figure S1** continued.

RRHRISSFRPVED

MW calc 1653.9 Da

YSKRTMTGYWSTQ

MW calc 1608.9 Da

**Figure S1** continued.

KRRRSSSYSPSPV

MW calc 1506.8 Da

DRRRSNTLDIMDG

MW calc 1548.9 Da

**Figure S1** continued.

SHGRTCSFGGFDL

MW calc 1383.7 Da

QRKRKLSFRRRTD

MW calc 1747.2 Da

**Figure S1** continued.

PHRRTLSFDTSKM

MW calc 1575.9 Da

FRKRLSTFRKHSL

MW calc 1676.2 Da

**Figure S1** continued.

FHTRFRSMLRNVL

MW calc 1677.1 Da

FRSRLDSYVRSLP MW calc 1596.0 Da

**Figure S1** continued.

MHSRHNSFDTVNT

MW calc 1545.6 Da

EIQRLDTFSYSTN

MW calc 1573.8 Da

**Figure S1** continued.

GRRRKYSLGRASR MW calc 1563.1 Da

QKKRRHSFEHVSL

MW calc1651.9 Da

**Figure S1** continued.

THQRVHTGTRPYM

MW calc 1583.7 Da

KRVRSSSFTFHIT

MW calc 1565.9 Da

**Figure S1** continued.

QKKRRHSFEHVS

MW calc 1651.9 Da

QRKRKLSFRRRTD

MW calc 1747.2 Da

**Figure S1** continued.

**Figure S2. Purification of phosphorylated AKT1 variants**. (A) Coomassie stained SDS-PAGE and (B) Western blot of the flow through (FT), washes, elutions, and final pooled product (P1) of the affinity chromatography used to purify pAKT1^T308^ from recombinant production in *E. coli*. ΔPH-AKT1 has an expected molecular weight of 45 kDa. We have previously published purification and mass spectrometry data to confirm phosphorylation status for each AKT1 variants used here (Balasuriya et al., 2018a; Balasuriya et al., 2018b; Balasuriya et al., 2020). Purified fractions of each phospho-variant are visualized on Coomassie stained SDS-PAGE: (C) pAKT1^T308^ (lane 1, ~90% purity) and pAKT1^S473^ (lane 2, >95% purity); (D) ppAKT1^T308,S473^ (>95% purity).

**Figure S3. Batch to batch consistency in AKT1 phospho-form activity across different preparations.** The apparent catalytic rate (*k_app_*, fmol_substrare_/min/pmol_enzyme_) of the three active AKT1 phospho-forms: pAKT1^S473^ (left panel), pAKT1^T308^, and ppAKT1^T308,S473^ (right panel) is shown here. All activity assays were conducted following the kinase assay protocol (see Methods) using 200 μM of the GSK-3β peptide (SGRPRTTSFAESCKP) as the substrate target for AKT1. The enzymes (Preparation 1) represented by the blue-striped bars were purified and utilized in this study while activity represented by the white bars (Preparation 2) were used for our previous work (Balasuriya et al., 2018a; Balasuriya et al., 2018b). Error bars show 2 standard deviations about mean for three independent enzyme reactions (n=3).

*
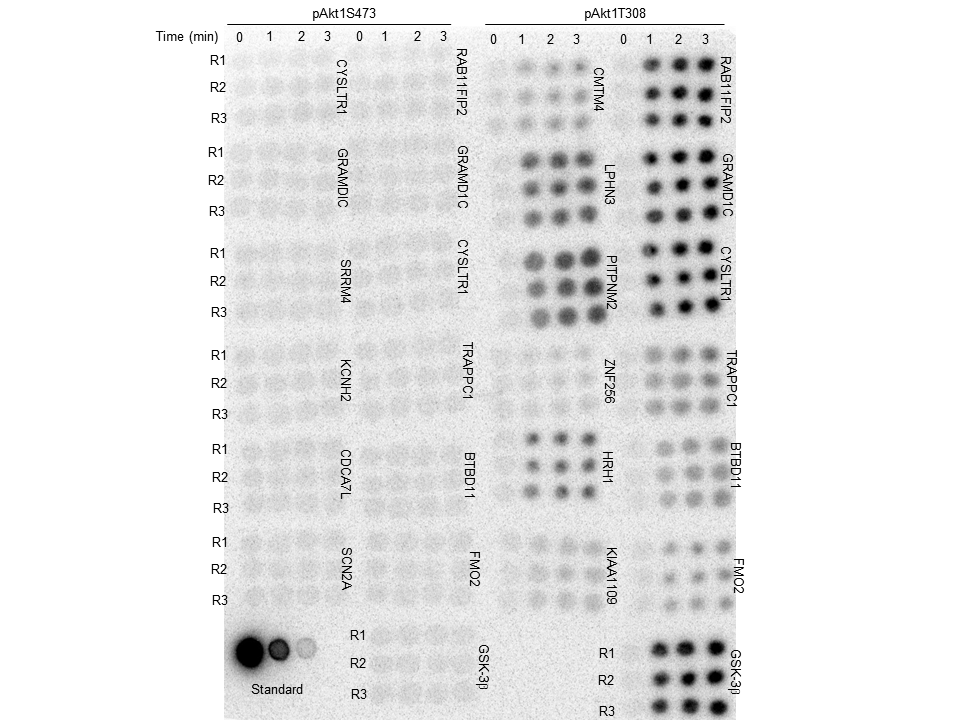
*

Figure S4. Radiograph detecting the presence of γ-[^32^P]-ATP in peptides phosphorylated by pAKT1^S473^ and pAKT1^T308^. Time courses (minutes) of the in vitro kinase assay catalyzed by the indicated phospho-form of AKT1 on the indicated peptide substrate. Each spot was generated by dispensing 3 μl of kinase assay reaction mixture onto P81 paper at the indicated time point after the initiation of the reaction. Assays were performed with three independent enzyme reactions (R1-R3). Three spots (Standard) correspond to the γ-[^32^P]-ATP standards which were used to calculate the relationship between spot intensity and [γ-[^32^P]-ATP].

******Figure S5. Radiograph detecting the presence of γ-[^32^P]-ATP in peptides phosphorylated by ppAKT1^S473,T308^.** Time courses (minutes) of the in vitro kinase assay catalyzed by the indicated phospho-form of AKT1 on the indicated peptide substrate. Each spot was generated by dispensing 3 μl of kinase assay reaction mixture onto P81 paper at the indicated time point after the initiation of the reaction. Assays were performed with three independent enzyme reactions (R1-R3) and 3 independent enzyme reactions. Three spots (Standard) correspond to the γ-[^32^P]-ATP standards which were used to calculate the relationship between spot intensity and the amount of γ-[^32^P]-ATP.

**
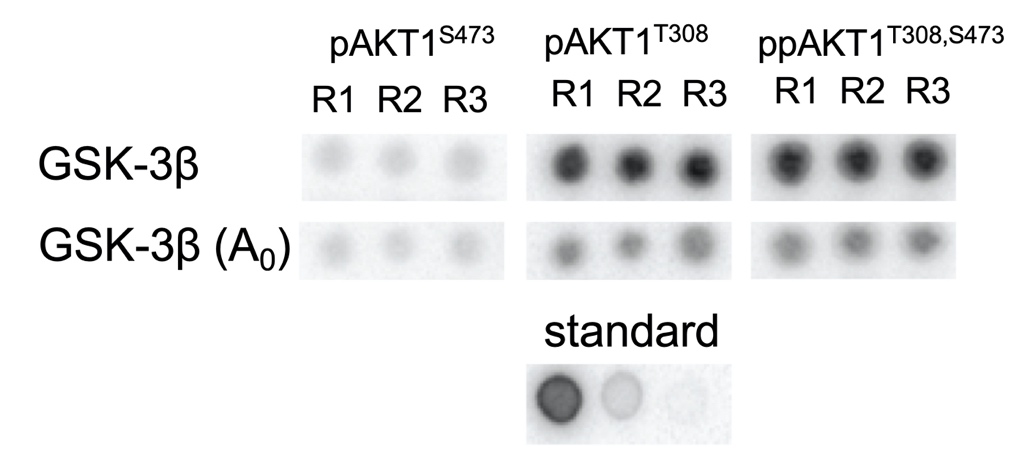
**

Figure S6. Radiographs that detect the presence of γ-[^32^P]-ATP in control peptides phosphorylated by pAKT1^S473^, pAKT1^T308^, and ppAKT1^S473,T308^. Spots of the in vitro kinase assay catalyzed by the indicated phospho-form of AKT1 on the indicated control peptide substrates. Assays were performed with three independent enzyme reactions (R1-R3). Each spot was generated by dispensing 3 μl of kinase assay reaction mixture onto P81 paper 10 minutes after the initiation of the reaction. Three spots (Standard) correspond to the γ-[^32^P]-ATP standards which were used to calculate the relationship between spot intensity and the amount of γ-[^32^P]-ATP.

**Figure S7. Initial velocities of each AKT1 phospho-forms with peptide substrates.** Initial velocities are given for pAKT1^S473^ (fmol of p-peptide/min) (A), pAKT1^T308^ (pmol of p-peptide/min) (B), ppAKT1^T308,S473^ (pmol of p-peptide/min) (C). Each bar represents the mean activity form 3 independent enzyme reactions containing a different peptide substrate. Raw data points are shown (open blue circles). All three AKT1 phospho-forms were tested using the known AKT1 substrate GSK-3β (SGRPRTTS_0_FAESCKP, red highlight) as a standard to assess the activity of the AKT1 preparations as well as a negative control variant of GSK-3β: GSK-3β (A_0_) (SGRPRTTA_0_FAESCKP, red highlight) to obtain minimal activity values for each phospho-form. Error bars represent 2 standard deviations about mean value for 3 independent enzyme reactions (n=3).

**Supplementary References**

Balasuriya, N., Davey, N.E., Johnson, J.L., Liu, H., Biggar, K.K., Cantley, L.C., et al. (2020). Phosphorylation-dependent substrate selectivity of protein kinase B (AKT1). *J Biol Chem* 295(24)**,** 8120-8134. doi: 10.1074/jbc.RA119.012425.

Balasuriya, N., Kunkel, M.T., Liu, X., Biggar, K.K., Li, S.S., Newton, A.C., et al. (2018a). Genetic code expansion and live cell imaging reveal that Thr308 phosphorylation is irreplaceable and sufficient for Akt1 activity. *J Biol Chem* 293(27)**,** 10744-10756. doi: 10.1074/jbc.RA118.002357.

Balasuriya, N., McKenna, M., Liu, X., Li, S.S.C., and O'Donoghue, P. (2018b). Phosphorylation-Dependent Inhibition of Akt1. *Genes (Basel)* 9(9). doi: 10.3390/genes9090450.
